# Identification of *Mycobacterium chimaera* in heater-cooler units in China

**DOI:** 10.1038/s41598-018-26289-5

**Published:** 2018-05-18

**Authors:** Xiaoxia Zhang, Ji Lin, Yu Feng, Xiaohui Wang, Alan McNally, Zhiyong Zong

**Affiliations:** 1 Center of Infectious Diseases, West China Hospital, Sichuan University, Chengdu, China; 2 Division of Infectious Diseases, State Key Laboratory of Biotherapy, Chengdu, China; 3 Center for Pathogen Research, West China Hospital, Sichuan University, Chengdu, China; 4 Department of Infection Control, West China Hospital, Sichuan University, Chengdu, China; 5 Institute of Microbiology and Infection, College of Medical and Dental Sciences, University of Birmingham, Birmingham, UK

## Abstract

A global outbreak of infections due to *Mycobacterium chimaera* has been linked to the LivaNova (formerly Sorin) 3 T heater-cooler units (HCUs). We performed a study to investigate *M. chimaera* from HCUs in China. Water samples were collected from all 3 T HCUs (n = 5) at our hospital in May 2017. Mycobacteria isolates were subjected to genome sequencing using the HiSeq X10 Sequencer. Species were identified based on average nucleotide identity with *M. chimaera* type strain DSM 44623^T^. Paired-end reads of all *M. chimaera* genomes were retrieved from the SRA database and, together with our isolates, were mapped against the chromosome of *M. chimaera* reference strain ZUERICH-1 to call SNPs. Mycobacteria grew from three HCUs manufactured in 2009 but not from the two in 2016. The three isolates were identified as *M. chimaera* and differed from each other by 4 to 6 SNPs, and from ZUERICH-1 by 7 to 10 SNPs. The three isolates belonged to the subgroup 1.1 and were most closely related to strains of the subgroup 1.1 from HCUs or patients in Europe, Australia/New Zealand and USA, suggesting the same common source. This is the first report of *M. chimaera* from HCUs in China.

## Introduction

*Mycobacterium chimaera*, a species of the *Mycobacterium avium* complex, is ubiquitous in the environment and is an opportunistic pathogen capable of causing human infections. In 2015, the infection control team at University Hospital Zurich reported that multiple patients had infections due to *M. chimaera* following open-heart surgey, with the outbreak linked to LivaNova (formerly Sorin) 3 T heater-cooler units (HCUs)^1^. Since then, outbreaks of infections due to *M. chimaera* among patients who received open-heart surgeries and were exposed to 3 T HCUs have also been reported in several European countries (Germany, the Netherland and UK), USA and Australia^2–4^. Infections due to *M. chimaera* are extremely difficult to treat and patients with such infections have a mortality rate of approximately 50%^4–6^. HCU-associated outbreaks of infections due to *M. chimaera* have been linked to the manufacturing site^7,8^. Although 3 T HCUs are also used in China, no such HCU-associated outbreaks have been reported. Here we report a study looking for the presence of *M. chimaera* in HCUs in a university hospital in China, with a hypothesis that the absence of such outbreaks is due to the absence of the organism in HCUs used in our hospital.

## Materials and Methods

### Settings

West China Hospital of Sichuan University, Chengdu, China, is a 5,000-bed tertiary hospital and serves as one of the major referral medical centers in western China. Around 2,600 patients received HCU-required open-heart surgeries in 2016 in the hospital. There are five 3 T HCUs, which are the only type of HCU, in the hospital. Three HCUs were manufactured in July 2009 and were introduced into the hospital in 2010 (n = 1) and 2012 (n = 2), while the remaining two were manufactured and were introduced into the hospital in August 2016. The HCUs were cleaned and disinfected every two to three months using the manufacturer’s protocol.

### Sampling and culture

We sampled 1 liter of water from each of the five 3 T HCUs at West China Hospital before they underwent disinfection in May 2017. The protocol developed by the European Center for Disease Prevention and Control (ECDC)^9^ was used for our study. Water samples were concentrated by filtration and were subjected to culture on M7H11 plates (Haibo, Qingdao, China) for up to eight weeks^9^.

### Whole genome sequencing

Mycobacteria isolates were subjected to whole genome sequencing with 300× coverage using the HiSeq X10 Sequencer (Illumina, San Diego, CA). The coverage was calculated based on the estimated genome size and the average output of the sequencer. Reads were trimmed using Trimmomatic^10^ and were then *de novo* assembled to contigs using the SPAdes program^11^ with careful mode turned on. Species identification of mycobacteria isolates was established based on average nucleotide identity (ANI) with the genome of strain DSM 44623^T^, the type strain of *M. chimaera* (GenBank accession no. LQOO00000000), using the JSpecies web program (http://imedea.uib-csic.es/jspecies/).

### Phylogenetic analysis

To determine the group and the subgroup of our three isolates, the paired-end reads of the isolates were mapped against the chromosome of the reference strain ZUERICH-1 of group 1 (Accession no. NZ_CP015272) using Bowtie 2^12^ and SAMtools^13^. Raw variants were called using Freebayes (https://github.com/ekg/freebayes). INDELs were discarded, and SNPs were filtered keeping only those with a minimum coverage, quality score and allele frequency of 10-fold, 30 and 0.75, respectively using vcflib (https://github.com/vcflib/vcflib). SNPs due to recombination were filtered using Gubbins^14^.

Illumina paired-end reads of all *M. chimaera* genomes available in the NCBI SRA database with a minimum of 200 Mbp (approximately 30× genome coverage) were retrieved (n = 508; for strains with multiple genome sequencing, only the first SRA record was retrieved). Ninety-eight of these genomes of 98 were discarded due to unresolvable problems (e.g. unequal length of sequence and the presence of invalid characters) of format and quality of their reads, resulting in a collection of 410 genome sequences. The raw sequence reads of these 410 strains were mapped against ZUERICH-1 and SNPs were called as described above. A phylogenetic tree of isolates belonging to subgroup 1.1 was constructed using RAxML^15^ with the GTRGAMMA model and a 1,000-bootstrap test.

#### Nucleotide sequence accession numbers

Draft whole-genome sequences of the three strains have been deposited into GenBank under the accession no. NTFV00000000, NTFW00000000 and NTFX00000000.

## Results

Mycobacteria grew from three HCUs manufactured in 2009 but did not grow from the two manufactured in 2016. One isolate from each of the three culture-positive HCUs, designated WCHMC000001, WCHMC000030 and WCHMC000032, was subjected to whole genome sequencing.

A total of 1.99 to 2.13 Gb clean bases and 6,618,824 to 7,116,445 clean reads were generated for the three isolates, which were then assembled to 6.1 to 6.6 Mb draft genomes with 84 to 112 contigs (77 to 104 were ≥1,000 bp in length) with a 67.46 to 67.64% GC content, respectively. The genome sequences of the three isolates had a 99.14 to 99.52% ANI with the genome of strain DSM 44623^T^. The three isolates were therefore identified as *M. chimaera*.

A recent study of the genomic sequence of 250 isolates revealed two major groups of *M. chimaera* and isolates of the HCU-related outbreaks belonged to group 1^7^. Using 10 single nucleotide polymorphisms (SNPs) for subgroup attribution, group 1 has been further classified into 11 distinct subgroups. Most isolates from water systems of 3 T HCUs in clinical use (Australia, Denmark, Germany, the Netherlands, New Zealand, UK, USA) and some from patients (Australia, Germany, the Netherlands, UK, USA) plus one from HCUs sampled at the LivaNova production site were clustered in the subgroup 1.1^7^. There were a maximum of 6 SNPs difference between our three isolates (Table 1), suggesting that they originated from a very recent common ancestor. WCHMC000001, WCHMC000030 and WCHMC000032 differed from ZUERICH-1 by only 7, 9 and 10 SNPs, respectively, suggesting that the three isolates belonged to group 1. Substitutions of guanine (G) by adenine (A) at positions 113,518 and 209,278 of the DSM 44623^T^ genome (GenBank accession no. LQOO00000000) were specific to subgroup 1.1^7^, both of which were found in our three isolates. This suggests that the three isolates belong to the subgroup 1.1.

**Table 1:** SNPs among the three isolates and most closely related isolates.

Strain (accession_country_source)	WCHMC000001 _China_HCU	WCHMC000030 _China_HCU	WCHMC000032 _China_HCU
SRR4068023_UK_HCU	2	4	5
SRR4324925_USA_HCU	2	4	5
ERR1463890_Germany_HCU	4	6	5
ERR1463906_Switzerland_HCU	4	6	5
ERR1463907_Germany_HCU	4	6	5
ERR1463911_Germany_HCU	4	6	5
ERR1463914_Netherlands_human	4	6	5
ERR1463929_Switzerland_HCU	4	6	5
ERR1464066_Switzerland_HCU	4	6	5
SRR4067862_UK_human	4	6	5
SRR4119598_UK_human	4	6	5
SRR4119619_UK_HCU	4	6	5
SRR4119623_UK_HCU	4	6	5
SRR4119626_UK_HCU	4	6	5
SRR4119636_UK_human	4	6	5
SRR4119656_UK_HCU	4	6	5
SRR4249865_NewZealand_HCU	4	6	5
SRR4249871_Australia_HCU	4	6	5
SRR4249872_Australia_HCU	4	6	5
SRR4249891_NewZealand_HCU	4	6	5

**Figure 1:**
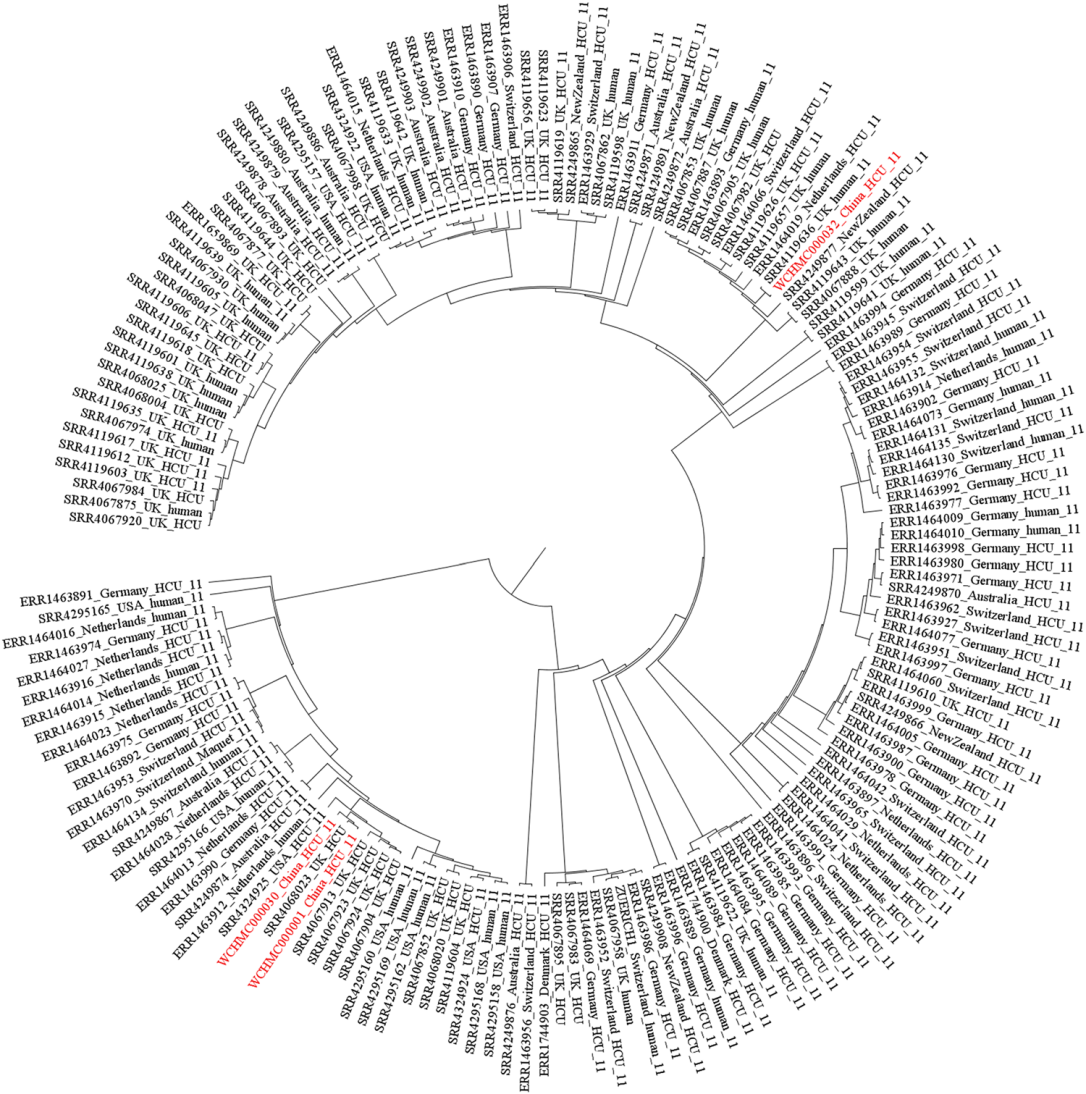
Maximum likelihood tree built from 295 SNP positions of the 169 strains of the subgroup 1.1 mapped to the genome of *M. chimaera* ZUERICH-1 shown as a circular phylogram. The three isolates in the present study were indicated in red.

Among the 410 strains from the SRA database, 169 were found to harbour either or both of the subgroup 1.1-specific SNPs. A total of 295 SNP sites were identified from these 169 strains. As revealed by the phylogenetic tree of isolates belonging to the subgroup 1.1 (Fig. 1), our three isolates were most closely related to strains recovered from HCUs or patients in several European countries, Australia, New Zealand and USA with a maximum of just 5 SNPs difference between them (Table 1). The high clonality of isolates of the subgroup 1.1 from different geographical regions suggests a common source, which is most likely from contamination at the manufacturer site.

## Discussion

We recovered *M. chimaera* from water tanks of three LivaNova 3 T HCUs at a university hospital. To be the best of our knowledge, this is the first report of *M. chimaera* from HCUs in China. Genome sequencing and phylogenetic analysis revealed that the three isolates were clonal and were placed within strains from multiple countries, suggesting the same common source.

It has been reported that brand new 3 T HCUs direct from the manufacturer can be quickly contaminated by *M. chimaera*^1,8^. However, no *M. chimaera* was recovered from the two recent HCUs manufactured in August 2016. There are several possible reasons. First, brand new 3 T HCUs have been shown to be contaminated after 158 to 358 days^16^. The two HCUs in our hospital had been in use for 8 months and it may be that sufficient time has not yet passed for them to be colonized to detectable levels by the organism. Second, the manufacturer has modified the post-production process in response to the findings of *M. chimaera* contamination^8^, which may reduce the risk of contamination in all new units. Third, the M7H11 media that we used has a higher detection limitation than the Mycobacterial Growth Indicator Tubes and may therefore be suboptimal^17^.

Unfortunately, there is no facility for blood culture of mycobacteria available in the hospital and there is no follow-up system for patients who received heart surgeries in the region. We are therefore unable to identify any clinical case of *M. chimaera* infection at present. Nonetheless, information of the global 3 T HCU-associated outbreak of infections due to *M. chimaera* and the local discovery of such *M. chimaera* strains has been disseminated to the cardiac surgeons and infectious disease physicians in the hospital. The facility for blood culture of mycobacteria will be established soon in the hospital to allow thorough future investigation of potential cardiac related *M. chimaera* infections.

Several measures have been proposed to minimize the risksof *M. chimaera* infections including strict separation of HCUs from the air volume of critical medical areas such as operating rooms, ensuring traceability of HCD use and following the updated manufacturer’s disinfection recommendations^5,18^. Unfortunately, it is not practical to place 3 T HCUs outside operating rooms in our hospital at present. The devices have been placed further away from patients, guided by smoking tests as described previously^19^. This practice could reduce patient risk but as patients are still exposed to HCUs in the room they need to be monitored for *M. chimaera* infections^18^. Strict separation of HCUs from operating rooms will be considered at the time of renovation. In addition, HCUs have been subjected to cleaning and disinfection using the updated protocol provided by the manufacturer (http://www.livanova.sorin.com/products/cardiac-surgery/perfusion/hlm/3t) since June 2017.

## References

[1] Sax H (2015). Prolonged Outbreak of *Mycobacterium chimaera* Infection After Open-Chest Heart Surgery. Clin Infect Dis.

[2] Achermann Y (2013). Prosthetic valve endocarditis and bloodstream infection due to *Mycobacterium chimaera*. J Clin Microbiol.

[3] Williamson D, Howden B, Stinear T (2017). *Mycobacterium chimaera* Spread from Heating and Cooling Units in Heart Surgery. N Engl J Med.

[4] Kohler P (2015). Healthcare-associated prosthetic heart valve, aortic vascular graft, and disseminated *Mycobacterium chimaera* infections subsequent to open heart surgery. Eur Heart J.

[5] Marra AR, Diekema DJ, Edmond MB (2017). *Mycobacterium chimaera* infections associated with contaminated heater-cooler devices for cardiac surgery: Outbreak Management. Clin Infect Dis.

[6] Chand M (2017). Insidious Risk of Severe *Mycobacterium chimaera* Infection in Cardiac Surgery Patients. Clin Infect Dis.

[7] van Ingen J (2017). Global outbreak of severe *Mycobacterium chimaera* disease after cardiac surgery: a molecular epidemiological study. Lancet Infect Dis.

[8] Haller S (2016). Contamination during production of heater-cooler units by *Mycobacterium chimaera* potential cause for invasive cardiovascular infections: results of an outbreak investigation in Germany, April 2015 to February 2016. Euro Surveill.

[9] European Center for Disease Prevention and Control. EU protocol for case detection, laboratory diagnosis and environmental testing of *Mycobacterium chimaera* infections potentially associated with heater-cooler units: case definition and environmental testing methodology. Stockholm, Sweden (2015).

[10] Bolger AM, Lohse M, Usadel B (2014). Trimmomatic: a flexible trimmer for Illumina sequence data. Bioinformatics.

[11] Bankevich A (2012). SPAdes: a new genome assembly algorithm and its applications to single-cell sequencing. J Comput Biol.

[12] Langmead B, Salzberg SL (2012). Fast gapped-read alignment with Bowtie 2. Nat Methods.

[13] Li H (2009). The Sequence Alignment/Map format and SAMtools. Bioinformatics.

[14] Croucher NJ (2015). Rapid phylogenetic analysis of large samples of recombinant bacterial whole genome sequences using Gubbins. Nucleic Acids Res.

[15] Stamatakis A (2014). RAxML version 8: a tool for phylogenetic analysis and post-analysis of large phylogenies. Bioinformatics.

[16] Schreiber PW (2016). Reemergence of *Mycobacterium chimaera* in heater-cooler units despite intensified cleaning and disinfection protocol. Emerg Infect Dis.

[17] Schreiber P. W. *et al*. Detection limit of *Mycobacterium chimaera* in water samples for monitoring medical device safety: insights from a pilot experimental series. *J Hosp Infect* In press: S0195-6701(17)30633*-*3 (2017). 10.1016/j.jhin.2017.11.007 29175077

[18] Sommerstein R (2017). *Mycobacterium chimaera* outbreak associated with heater-cooler devices: piecing the puzzle together. Infect Control Hosp Epidemiol.

[19] Sommerstein R (2016). Transmission of *Mycobacterium chimaera* from heater-cooler units during cardiac surgery despite an ultraclean air ventilation system. Emerg Infect Dis.

